# Supplementary material for: Prophage-triggered membrane vesicle formation through peptidoglycan damage in Bacillus subtilis
Source: Nat Commun. 2017 Sep 7;8:481. doi: 10.1038/s41467-017-00492-w (PMC5589764; doi:10.1038/s41467-017-00492-w)
Supplement: Supplementary file 1 — Supplementary Information [file 41467_2017_492_MOESM1_ESM.pdf]

Description of Supplementary Files

File Name: Supplementary Information
Description: Supplementary Tables, Supplementary Figures and Supplementary References

File Name: Supplementary Movie 1
Description: MVs are released by B. subtilis 168 at the onset of the stationary phase. Cell membrane was stained with FM4-64. Dead cells and extracellular DNA are stained with SYTOX green (green).

File Name: Supplementary Movie 2
Description: MV formation in induced B. subtilis 168 (P_{xylA}-xhlAB-xlyA) cells. Cells were stained with FM4-64 and inoculated on LB agarose pads containing 0.1% xylose and fluorescent dyes and mounted for live cell imaging by CLSM. Membranes were stained with FM4-64 (red), dead cells and extracellular DNA are stained with SYTOX green (green).

File Name: Supplementary Movie 3
Description: Close up image of MV formation in B. subtilis 168 (P_{xylA}-xhlAB-xlyA). MV formation was induced and observed as described in Supplemental movie 2.

File Name: Supplementary Movie 4
Description: ECT imaging of MV formation. Shown are different 2D slices and a model of the cell shown in Fig. 3d. Peptidoglycan, white; inner membrane, orange.

File Name: Supplementary Movie 5
Description: Cell death triggers MV release in neighboring cells. B. subtilis 168 cells were stained with FM4-64 and then incubated for 5h at 37 °C on an LB agarose pad containing SYTOX green (green) and FM4-64 (red). The fate of cells was monitored by CLSM.

File Name: Peer Review File

Supplementary Table 1: Strains and plasmids used in this study

Name	Genotype or description	Source or reference
***B. subtilis* strains**		
168	Wild-type *trpC2*	Laboratory stock
MS	*SPβ PBSX skin* λPr-neo::Δ*upp trpC2*	NBRP collection
MGB469	*SPβ PBSX pro1-6 skin pks pps*	NBRP collection
Δ*blyA-bhlAB*	*trpC2 blyA-bhlA-bhlB*::*erm*	This study
Δ*xhlAB-xlyA*	*trpC2 xhlA-xhlB-xlyA*::*spc*	This study
Δ*blyA-bhlAB*Δ*xhlAB-xlyA*	*trpC2 blyA-bhlA-bhlB*::*erm* *xhlA-xhlB-xlyA*::*spc*	This study
168 (P_{xylA})	*trpC2 amyE*::*xylR*, P_{xylA}, *cm*	This study
168 (P_{xylA}-*blyA-bhlAB*)	*trpC2 amyE*::*xylR*, P_{xylA}-*blyA-bhlA-bhlB, cm*	This study
168 (P_{xylA}-*xhlAB-xlyA*)	*trpC2 amyE*::*xylR*, P_{xylA}-*xhlA-xhlB-xlyA, cm*	This study
168 (P_{xylA}-*xhlB-xlyA*)	*trpC2 amyE*::*xylR*, P_{xylA}-*xhlB-xlyA, cm*	This study
168 (P_{xylA}-*xlyA*)	*trpC2 amyE*::*xylR*, P_{xylA}-*xlyA, cm*	This study
Δ*recA*	*trpC2 recA*::*cm*	This study
amyE::recA	*trpC2 amyE*::*recA*, *kan*	This study
amyE::kan	*trpC2 amyE*::*kan*	This study
Δ*recA amyE::recA*	*trpC2 recA*::*cm amyE*::*recA, kan*	This study
Δ*recA amyE::kan*	*trpC2 recA*::*cm amyE*::*kan*	This study
Δ*ponA*	*trpC2 ponA*::*kan*	This study
Δ*ponA* (P_{xylA}-*xhlAB-xlyA*)	*xhlAB-xlyA* inducible strain of Δ*ponA*	This study
TAY3000	*trpC2 lys1 aprEΔ3 nprE18 nprR2 amyE*::*kan*	1
TMO310	*trpC2 aprE*::*lacI Pspac-mazF, spec*	2
***E. coli* strains**		
JM109	*recA*1 *endA*1 *gyrA*96 *thi*-1 *hsdR*17(r_K^- m_K^+) *e*14$^-$ (*mcrA*$^-$) *supE*44 *relA*1 Δ(*lac-proAB*) F'〔*traD*36 *proAB*$^+$ *lac* I^q *lacZ*ΔM15〕	Takara Bio

Mach1	*F– Φ80lacZΔM15 ΔlacX74 hsdR(rK–, mK+) ΔrecA1398 endA1 tonA*	Thermo Fisher Scientific
**Plasmid**		
pMutinNC	Integration vector, *amp*, *erm*	3
pDH88	Integration vector, *amp*, *cat*	4
pEGFP	Plasmid harboring *egfp*	Clontech
pZsGreen	Plasmid harboring *zsGreen*	Clontech
pHY300PLK	*E. coli-B. subtilis* shuttle vector, *amp*, *tet*	Takara Bio
pHY300-P*veg*-*egfp*-term	P*veg*-*egfp* in pHY300PLK	This study
pHY300-P$_L$-*egfp*-term	P$_L$-*egfp* in pHY300PLK	This study
pHY300-*egfp*-term	Promoterless *egfp* in pHY300PLK	This study
pHY300-P*veg*-ZsGreen-term	P*veg*-*zsGreen* in pHY300PLK	This study
pHY300-P$_L$-ZsGreen-term	P$_L$-*zsGreen* in pHY300PLK	This study
pHY300-ZsGreen-term	Promoterless *zsGreen* in pHY300PLK	This study

Note: NBRP (National BioResource Project, Japan)

Supplementary Table 2: Primers used in this study

Primer ID	Primer sequence (5' to 3')
TYP1	TTCAAAACCTCTTTACTGCCGTTATTCGC
TYP2	GGAGTGTCAAGAATGTTTGCAAAACGATTC
TYP3	CACGTAATCAAAGCCAGGCTGATTCTGACC
TYP4	TCAATGGGGAAGAGAACCGCTTAAGCCC
TYP7	GGCCGGATCCAAGAGTTTGTAGAAACGCAA
TYP8	GGCCGAATTCCGATAAACCCAGCGAACCAT
TYP20	GGCCGAATTCGAGTGCAACTAAGTTTGAAA
TYP57	AATACGCTCCACTTCATAACATGG
TYP58	ACTGTTGAATGTGCACAAGCAG
TYP59	TCTCTTTCTTTGGCACAATGGCTAG
TYP60	CACTCCACAATATTGAACACGTCC
TYP61	GAAATTAAGGAGATGTTTTTTCTAGCACAAAAGAAAAACG
TYP62	CGTTTTTCTTTTGTGCTAGAAAAAACATCTCCTTAATTTC
TYP63	TTGCGTTTCTACAAACTCTTAAAAACAATTACATAACTGC
TYP64	GCAGTTATGTAATTGTTTTTAAGAGTTTGTAGAAACGCAA
TYP65	GGGAATCTTCAGCAATCTGAGTTTG
TYP66	GCAGCAGTTAATGTCGCAGCAGGC
TYP67	TGACGTCTGACAGCATTGTCACG
TYP68	AATACGGAAAAGTGGTTCTCATCC
TYP69	ATGTGAAGGAGGAGTGAGAGCAAAAATTATATGGAGATCT
TYP70	AGATCTCCATATAATTTTTGCTCTCACTCCTCCTTCACAT
TYP71	ACTTTAATTTAGTGAAGCTTTCAAAGACCATAAAAATCCC
TYP72	GGGATTTTTATGGTCTTTGAAAGCTTCACTAAATTAAAGT
TYP81	TGCGTTTCTACAAACTCTTTGCTTCAGAAATACTCCTAGA
TYP82	CTAGGAGTATTTCTGAAGCAAAGAGTTTGTAGAAACGCAA
TYP83	TTGAATGAATTTATTTTTAAAAAATAACCAAAAAGCAAGG
TYP84	CCTTGCTTTTTGGTTATTTTTTAAAAATAAATTCATTCAA
TYP85	TTGAATGAATTTATTTTTAAAATTAGAAATTAAGGAGATG
TYP86	CATCTCCTTAATTTCTAATTTTAAAAATAAATTCATTCAA

TYP87	AAGAGAACTTGTTTAAATAGAAAATAACCAAAAAGCAAGG
TYP88	CCTTGCTTTTTGGTTATTTTCTATTTAAACAAGTTCTCTT
TYP89	TTGAATGAATTTATTTTTAATCTGCATGTGAAGGAGGAGT
TYP90	ACTCCTCCTTCACATGCAGATTAAAAATAAATTCATTCAA
TYP91	TCGCAGCGCAATTAAGCTGAAAAATAACCAAAAAGCAAGG
TYP92	CCTTGCTTTTTGGTTATTTTTCAGCTTAATTGCGCTGCGA
TYP93	TTGAATGAATTTATTTTTAAGCTGCAGCATTAAAGGGGGA
TYP94	TCCCCCTTTAATGCTGCAGCTTAAAAATAAATTCATTCAA
TYP101	TTGAATGAATTTATTTTTAATCTGACGAAATAAGGAGAGA
TYP102	TCTCTCCTTATTTCGTCAGATTAAAAATAAATTCATTCAA
TYP105	TAATGTCACTAACCTGCCCCGTTTAGGCTGGGCGGTGATA
TYP106	TATCACCGCCCAGCCTAAACGGGGCAGGTTAGTGACATTA
TYP107	GGCCGAATTCGAAAATTGGATAAAGTGGGA
TYP108	TAGCGGTGCCTCAAAATTATTTGC
TYP109	TTTCTGCGGCAGAAAGCTTTACC
TYP110	GTAGTGTTTACGGTCAGCATAGCC
TYP111	AATACGTGCTTACCGGATCACTTG
TYP112	ATAAAGGAGGAAAAAATAGAGAAAATTGGATAAAGTGGGA
TYP113	TCCCACTTTATCCAATTTTCTCTATTTTTTCCTCCTTTAT
TYP114	TAATGTCACTAACCTGCCCCAAATAAAATAAGTTTCAAAT
TYP115	ATTTGAAACTTATTTTATTTGGGGCAGGTTAGTGACATTA
TYP116	GGCCGGATCCAGAAAACGCGGCGCTAAATA
TYP117	GGCCGAATTCCAATATTTTTATCATTTTTTTGCAAAATAC
TYP118	GGCCGAATTCTCGTTTTAAAAACCCCTGCC
TYP119	ATAGTGTTGGCCAGCAAGAGGCGA
TYP120	CTTGCTGGCCAACACTATTTTGAATGCGTACAGACATTCTAAGC
TYP121	CTCGCCCTTGCTCACCATTGCATCCACCTCACTACATTTATTGTAC
TYP122	CCTTGCTTTTTGGTTATTTTTTACTTGTACAGCTCGTCCA
TYP123	TGGACGAGCTGTACAAGTAAAAAATAACCAAAAAGCAAGG
TYP124	GGCCGGATCCGAGTGCAACTAAGTTTGAAAAATCAG
TYP125	ATGGTAGCAAGGGCGAGGAGC
TYP126	CTTGCTGGCCAACACTATAAGCTTGTCATACGTTTGCCAC

TYP127	TCACCACCTTTTCCCTATATAAAATTAGAAGCTTTTGACGTAATACAGGC
TYP128	GCTGGCCAACACTATATGGTGAGCAAGGGCGAGGAGC
TYP129	GGAAAAGGTGGTGAACTACTATGGCTCAGTCAAAGCACGG
TYP130	CCTTGCTTTTTGGTTATTTTTCAGGGCAATGCAGATCCGG
TYP131	CTTTTTGGTTATTTTTCAGGGCAATGCAGATCCGG
TYP132	AGGTGGTGAACTACTATGACCATGATTACGCCAAGCTTGC
TYP133	CGGGTTTCGCCACCACTGATTTGAGCGTCA
TYP134	AACATTCTCAAAGGGATTTCTAAATCGTTA
TYP135	CACCTTTTCCCTATATAAAATACATTTATTGTACAACACG
TYP136	CGTGTTGTACAATAAATGTATTTTATATAGGGAAAAGGTG
TYP137	CACCTTTTCCCTATATAAAAATAGTGTTGGCCAGCAAGAG
TYP138	CTCTTGCTGGCCAACACTATTTTTATATAGGGAAAAGGTG
TAY139	AGTAATCGCGGAATGACCCTCG
TAY140	CCTCGAAGATGACTTAAACGAAACG
TAY141	GTGTTGGCTTCCTATGAGCAATAC
TAY142	GTTCCTCGGATATGAAGAAAACC
TAY143	TAACGAAAGGTTGAGATGTTCGATAAACCCAGCGAACCAT
TAY144	ATGGTTCGCTGGGTTTATCGAACATCTCAACCTTTCGTTA
TAY145	TTTAGATGTCTAAAAAGCTTACAAAAAAGCCGTCACCTTT
TAY146	AAAGGTGACGGCTTTTTTGTAAGCTTTTTAGACATCTAAA

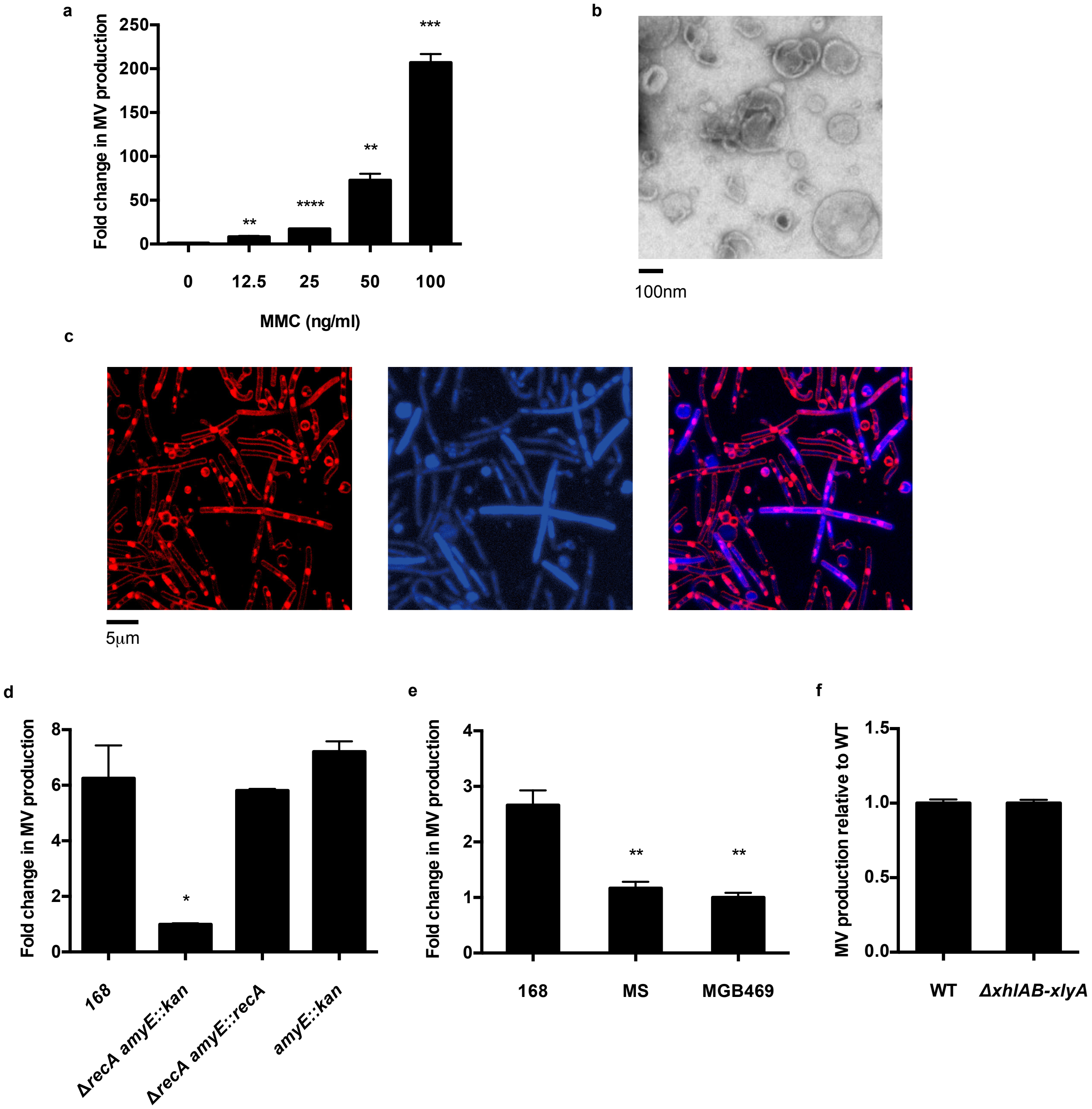

Supplementary Figure 1. MV production in *B. subtilis* is induced by MMC.
(a) MV production relative to the uninduced control is shown. MMC was added at the concentrations indicated in the figure when cultures reached an OD_{600} of 0.3-0.4. The cultures were incubated for 4h before MVs were quantified. *n*=3; mean±s.d. ***P* < 0.01, ****P* < 0.001, *****P* < 0.0001 (unpaired t-test with Welch's correction). **(b)** TEM images of MVs purified from the supernatant of *B. subtilis* cultures treated with MMC. **(c)** CLSM images of *B. subtilis* cells treated with 5ng/mL MMC. Cells were stained with the membrane stain FM4-64 (red) and the DNA stain DAPI (blue). **(d and e)** Effect of *recA* and prophages on MMC-induced MV production relative to the untreated control is shown. The *recA* mutant was complemented by insertion of the wild-type allele in the *amyE* gene region. An *amyE*::kan insertion mutant was used as a control. Strain MS lacks SPβ, PBSX and SKIN prophage sequences. MGB469 lacks all prophage sequences except *pro*Φ *7*. *n*=3; mean±s.d. **P* < 0.05, ***P* < 0.01 (unpaired t-test with Welch's correction). **(f)** MV production relative to the wild-type under MMC non-added conditions. Cells were cultured in LB medium for 24h at 37 °C before MVs were quantified. *n*=3; mean±s.d.

a

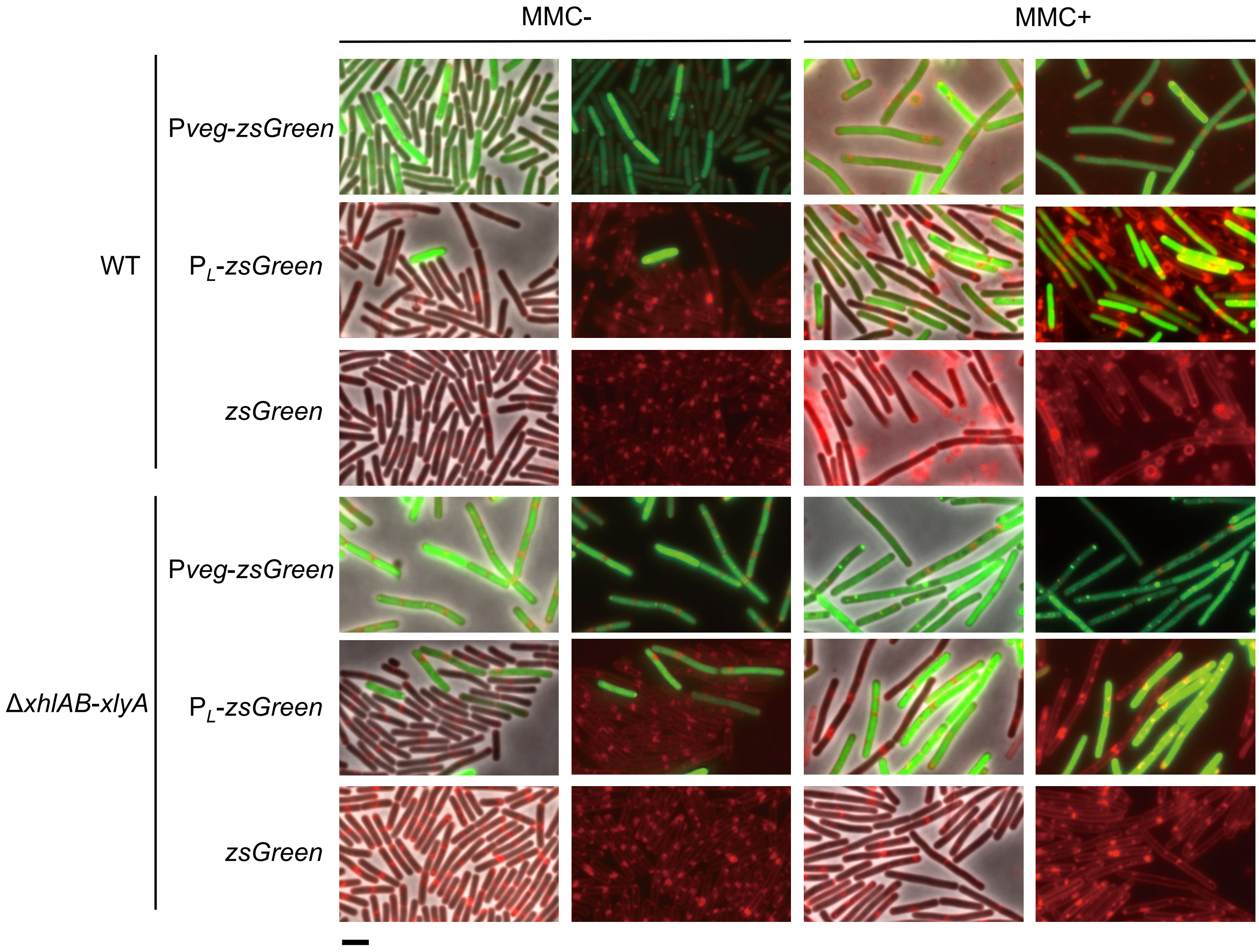

b

Strain	Treatment	% of cells with fluorescence (n)		
		P*veg-zsGreen*	P$_L$-*zsGreen*	*zsGreen*
WT	Untreated	99.8 ± 0.10	2.45 ± 0.32	0.03 ± 0.01
	MMC	99.8 ± 0.05	83.0 ± 2.03	0.08 ± 0.03
Δ*xhlAB-xlyA*	Untreated	100 ± 0.03	5.22 ± 0.50	0.06 ± 0.02
	MMC	99.7 ± 0.05	83.8 ± 1.16	0.13 ± 0.02

Supplementary Figure 2. The PBSX phage is expressed in a subpopulation.
(a) Activity of the P$_L$ late operon promoter of PBSX. The P*veg* promoter was used as a positive control and *zsGreen*, which lacked a promoter, was used as a negative control. Following addition of MMC, cultures were incubated for 1h before pictures were taken. Panels show phase contrast images merged with ZsGreen (green) and the membrane strain FM4-64 (red) (columns 1 and 3), and FM4-64 merged with ZsGreen (columns 2 and 4). The merged image of P$_L$-*zsGreen* and FM4-64 is also shown in Fig. 2c. Bar, 5μm. **(b)** Proportion of cells expressing transcriptional fusions of the respective promoter regions. Fluorescence of each cell was measured using a flow cytometer. *n*=3; mean±s.e. (unpaired t-test with Welch's correction).

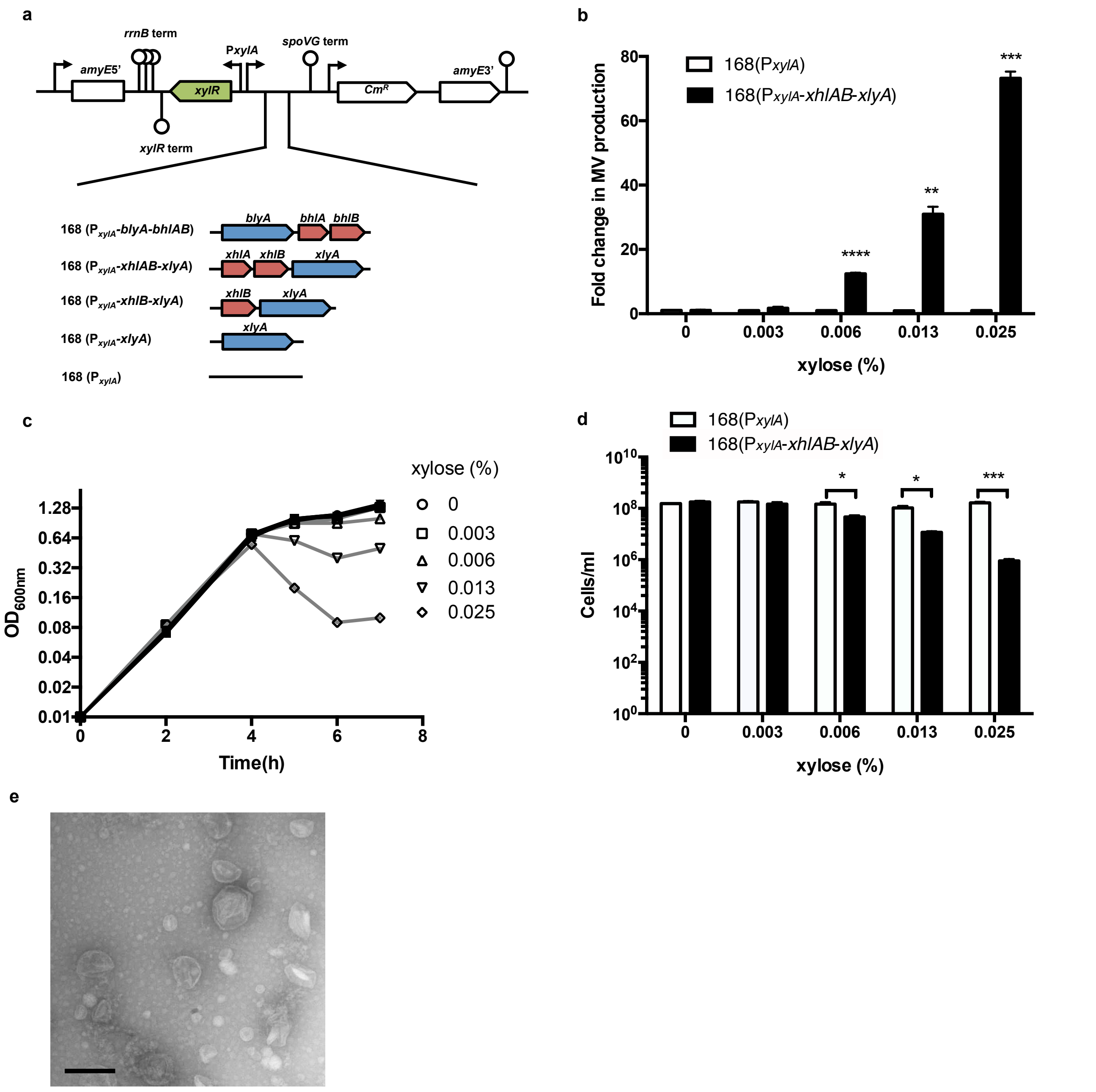

Supplementary Figure 3. The PBSX holin-endolysin system stimulate MV biogenesis in *B. subtilis* 168. (a) Construction of holin-endolysin conditional strains. Different genes of the PBSX holin-endolysin locus were placed under the control of the xylose-inducible promoter P_{xylA} and the resulting gene cassettes were inserted into the *amyE* locus. The control strain 168 (P_{xylA}) has no gene downstream of the *xylA* promoter. **(b)** MV production is stimulated by the expression of *xhlAB-xlyA.* Gene expression was induced by adding various amounts of xylose to cultures of strain 168 (P_{xylA}-*xhlAB-xlyA*). MV production relative to the uninduced control is shown. *n*=3; mean±s.d. ***P* < 0.01, ****P* < 0.001, *****P* < 0.0001 (unpaired t-test with Welch's correction). MV production is not induced by xylose in the control strain 168 (P_{xylA}). **(c and d)** Growth curve and CFUs of the *xhlAB-xlyA* inducible strain 168 (P_{xylA}-*xhlAB-xlyA*) and the control strain 168 (P_{xylA}). The mean of two independent assays are shown for the growth curve. Black lines indicate the control strain 168 (P_{xylA}) and grey lines indicate strain 168 (P_{xylA}-*xhlAB-xlyA*). CFUs were determined after 7h (*n*=3; mean±s.e.m. **P* < 0.05, ****P* < 0.001 (unpaired t-test)). **(e)** TEM image of MVs purified from the supernatant of an induced *B. subtilis* 168 (P_{xylA}-*xhlAB-xlyA*) culture. Bar, 500nm.

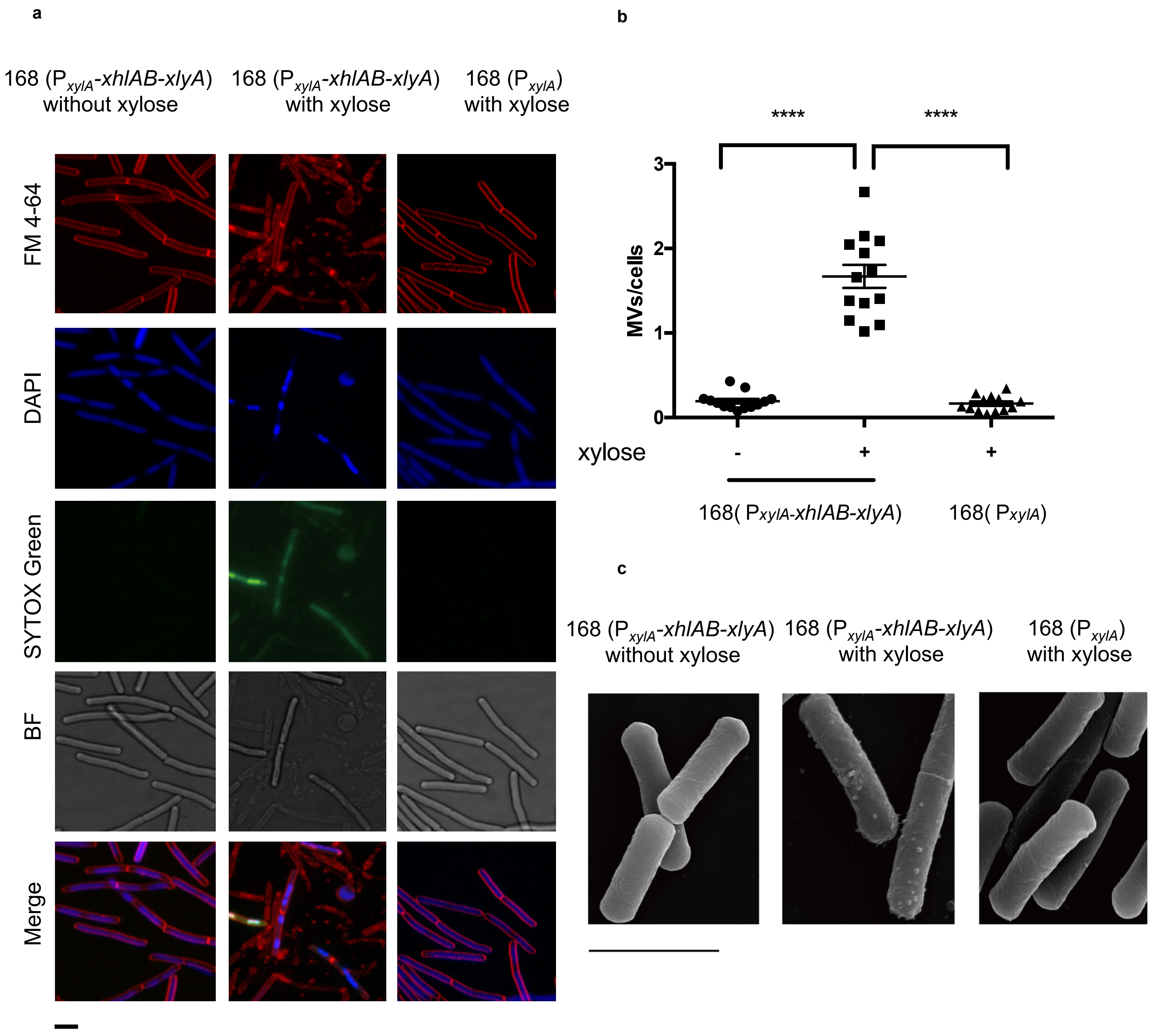

Supplementary Figure 4. MV production is induced by expression of the *xhlAB-xlyA* genes. (a) CLSM images of induced and uninduced 168 (P_{xylA}-*xhlAB-xlyA*) cells, and the control strain 168 (P_{xylA}). Membranes were stained with FM4-64 (red), DNA with DAPI (blue) and dead cells with SYTOX green (green). Bar, 5μm. **(b)** Quantification of MV-producing cells after staining of samples with FM4-64 (red). ****$P < 0.0001$ (unpaired t-test with Welch's correction). n=13; mean±s.e.m. **(c)** SEM images of the same samples. Bar, 2μm.

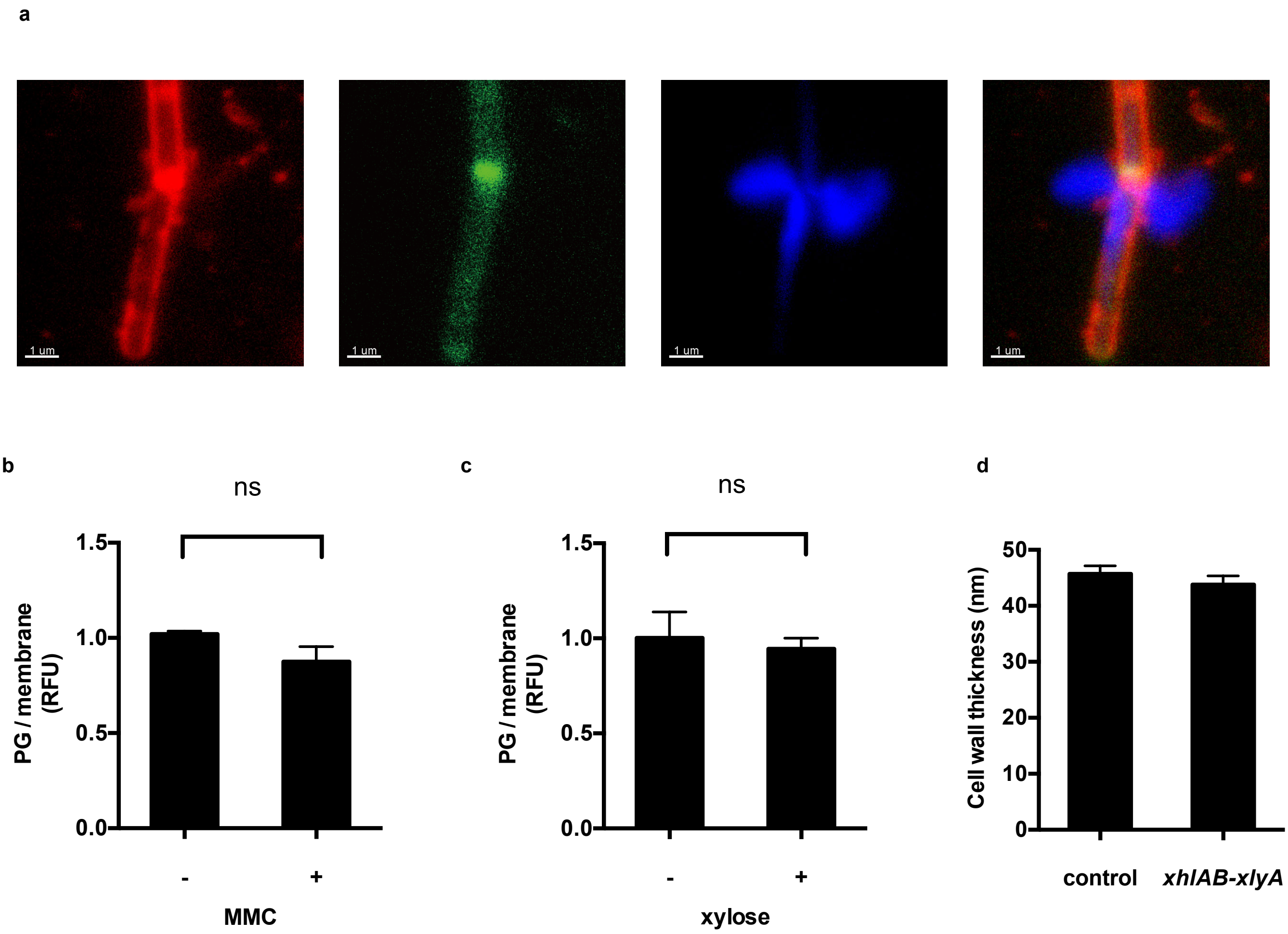

Supplementary Figure 5. PG is only partially degraded by the XhlAB-XlyA holin-endolysin system. **(a)** CLSM images of induced 168 (P_{xylA}-*xhlAB-xlyA*) cells. Membranes are shown in red, PG in green and DNA in blue. Bar, 1μm. **(b and c)** Amount of PG relative to the amount of membrane material in induced and uninduced cultures. MV production was induced either by adding MMC to wild-type (WT) cultures (b) or by adding xylose to strain 168 (P_{xylA}-*xhlAB-xlyA*) (c). Cells were harvested and washed twice in PBS before FDL and FM4-64 fluorescence was quantified. n=3; mean±s.d. (unpaired t-test with Welch's correction). **(d)** The overall thickness of the cell wall remains unchanged when the holin-endolysin system is induced. Cell wall thickness was measured using the thin section TEM images of induced *B. subtilis* 168 (P_{xylA}-*xhlAB-xlyA*) and the control strain 168 (P_{xylA}). n=30; mean±s.e.m.

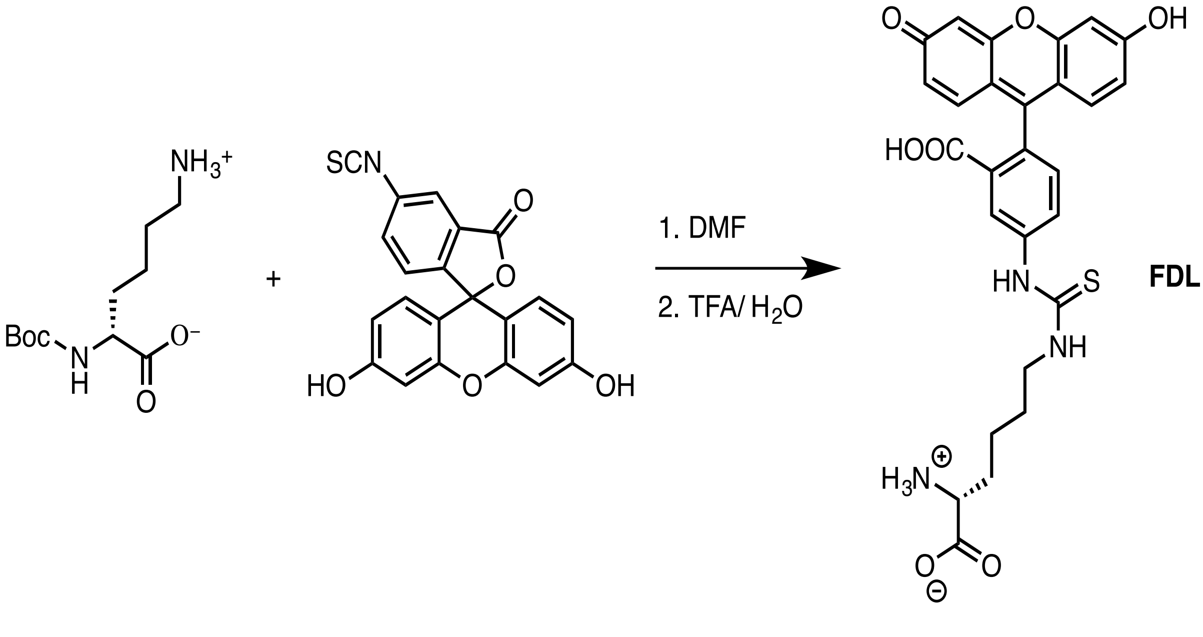

FDL

Supplementary Figure 6. Procedure for the preparation of FDL. The details are given in the method section of the manuscript.

Supplementary References

1 Yamamoto, T. *et al.* SP10 infectivity is aborted after bacteriophage SP10 infection induces nonA transcription on the prophage SPbeta region of the *Bacillus subtilis* genome. *J. Bacteriol.* **196**, 693-706, doi:10.1128/JB.01240-13 (2014).

2 Morimoto, T., Ara, K., Ozaki, K. & Ogasawara, N. A new simple method to introduce marker-free deletions in the *Bacillus subtilis* genome. *Genes Genet. Syst.* **84**, 315-318 (2009).

3 Morimoto, T. *et al.* Six GTP-binding proteins of the Era/Obg family are essential for cell growth in *Bacillus subtilis*. *Microbiology* **148**, 3539-3552, doi:10.1099/00221287-148-11-3539 (2002).

4 Henner, D. J. Inducible expression of regulatory genes in *Bacillus subtilis*. *Methods Enzymol.* **185**, 223-228 (1990).
